# Isotope-Dilution Gas Chromatography-Mass Spectrometry Method for the Selective Detection of Nicotine and Menthol in E-Cigarette, or Vaping, Product Liquids and Aerosols

**DOI:** 10.3389/fchem.2021.754096

**Published:** 2021-09-27

**Authors:** José J. Pérez, Clifford H. Watson, Benjamin C. Blount, Liza Valentín-Blasini

**Affiliations:** Tobacco Products Laboratory, Tobacco and Volatiles Branch, Division of Laboratory Sciences, National Center for Environmental Health, U.S. Centers for Disease Control and Prevention, Atlanta, GA, United States

**Keywords:** e-cigarettes, vapes, nicotine, menthol, gas chromatography-mass spectrometry

## Abstract

We developed a quantitative method for analyzing nicotine and menthol in e-cigarette, or vaping, products (EVPs). These products may adversely impact health through inhalational exposure to addictive and harmful chemicals. The presence of unknown substances in do-it-yourself e-liquids, counterfeits, or unregulated products may increase exposure to harmful chemicals, as underscored by the 2019 EVP use-associated lung injury (EVALI) outbreak. To minimize these risks, it is important to accurately quantify nicotine and menthol in e-liquids and aerosol emissions to evaluate EVP authenticity, verify product label accuracy, and identify potentially hazardous products. We developed a simple, versatile, high-throughput method using isotope-dilution gas chromatography-mass spectrometry for quantifying nicotine and menthol concentrations in both e-liquid contents and machine-generated aerosol emissions of EVPs. Rigorous validation has demonstrated that the method is specific, precise (CV<2.71%), accurate (percent error ≤7.0%), and robust. Linear calibration ranges from 0.01 to 1.00 mg/ml for both analytes was achieved, corresponding to expected analyte levels in e-liquids and machine-generated EVP aerosols. Limits of detection (LODs) in the final 10-ml sample extract were 0.4 μg/ml for nicotine and 0.2 μg/ml for menthol. The method was used to analyze aerosol emissions of 141 EVPs associated with the 2019 EVALI outbreak; detectable levels of nicotine (2.19–59.5 mg/g of aerosol) and menthol (1.09–10.69 mg/g of aerosol) were observed in 28 and 11%, respectively, of the samples analyzed. Nicotine was not detected in any of the tetrahydrocannabinol (THC), cannabidiol (CBD), or oil-based products, while menthol (2.95 mg/g of aerosol) was only detected in one of these products (THC-labeled). The analytical method can be used to quantify nicotine and menthol concentrations in the e-liquids and aerosols from a range of EVPs, and these findings highlight a difference between e-cigarette and other vaping products.

## Introduction

E-cigarette, or vaping, product (EVP) use has increased substantially in the United States over the last few years, both for traditional atomizer/cartomizer e-cigarettes designed to deliver nicotine (Wang et al., 2020) and for more recent ceramic-cell vape products designed to deliver cannabinoids (Knapp et al., 2019). These increases are likely driven by a number of factors and are primarily impacting youth and young adults (King et al., 2020). The 2019 U.S. e-cigarette, or vaping, product use-associated lung injury (EVALI) outbreak (Centers for Disease Control and Prevention Office on Smoking and Health, 2019) underscored the diversity of products used by many EVALI case patients (Trivers et al., 2021). Furthermore, many young EVP users tend to experiment with products from unregulated/online vendors as well as do-it-yourself (DIY) liquids (Cox et al., 2019). These unregulated products have unknown health consequences and their chemical ingredients—including their origin, quality, and safety—may also be unknown. For example, the EVALI outbreak was strongly associated with vitamin E acetate which was being used as a diluent in EVPs (Blount et al., 2020; Krishnasamy et al., 2020; Puetz et al., 2021). Other chemical constituents in these unregulated products may also contribute to adverse health outcomes.

Analytical methods are needed to measure nicotine and menthol accurately and precisely in different types of e-liquids, vape liquids, and EVP aerosols. Nicotine is the primary addictive chemical in tobacco products and its accurate quantitation is critical for establishing product authenticity, verifying product-label accuracy, and assessing addiction potential of a product. Menthol, which is used as a tobacco product flavor additive (U.S. Food and Drug Administration, 2021a), may contribute to adverse health outcomes by altering users’ smoking behavior (Watson et al., 2017) and the U.S. Food and Drug Administration has declared its intention to ban its use in cigarettes (U.S. Food and Drug Administration, 2021b). Various methods have been described to measure nicotine concentrations in traditional atomizer/cartomizer devices and in hydrophilic solvents such as propylene glycol (PG) and glycerol (GLY) (Trehy et al., 2011; Goniewicz et al., 2013; Famele et al., 2015; Lisko et al., 2015; Ogunwale et al., 2017; Gholap et al., 2018; Reilly et al., 2020). However, these existing methods have not been validated for measuring nicotine and menthol in hydrophobic, oil-based liquids and aerosols that can result from DIY mixing of ingredients and from using different types of liquids in the same device. We describe the development, validation, and application of a new, simple, sensitive, high-throughput, and selective isotope-dilution gas chromatography-mass spectrometry (ID-GC-MS) method for the simultaneous quantitation and characterization of nicotine and menthol in e-liquids and machine-generated aerosol emissions of EVPs. This method was used to quantitatively analyze aerosol emissions from 141 EVPs associated with the 2019 EVALI outbreak. To enable analysis of a broader range of EVPs with differing chemical compositions, the new method was validated for quantitative analysis in both hydrophilic and hydrophobic e-liquid matrices. This is the first report of these analytes quantitatively measured in samples from the EVALI response.

## Materials and Methods

### Chemicals and Materials

(−)-Nicotine [CAS# 54-11-5; ≥99% (GC), liquid] and isotopically labeled (±)-nicotine-(pyridine-d_4_) internal standard (ISTD; CAS# 350818-69-8; isotopic purity: ≥98 atom% D, ≥98% chemical purity) were purchased from Sigma-Aldrich (St. Louis, MO, United States). L (−)-menthol (CAS# 2216-51-5; 99.5%; category 1 standard), PG (CAS# 57-55-6; ≥99.5%; USP/FCC), GLY (CAS# 56-81-5; ≥99.5%; certified ACS), and methanol (MeOH; CAS# 67-56-1; ≥99.9%; HPLC grade) were purchased from Thermo Fisher Scientific (Waltham, MA, United States). The isotopically labeled (−)-menthol-(1,2,6,6-d_4_) ISTD (98%) was obtained from Cambridge Isotope Laboratories (Andover, MA, United States). Research grade helium was purchased from Airgas Inc. (Hapeville, GA, United States).

Cambridge filter pads (CFPs; 44-mm) for collecting machine-generated EVP aerosol emissions were purchased from Thermo Fisher Scientific (Waltham, MA, United States). CFP holders were purchased from Cerulean (Molins PLC, Milton Keynes, United Kingdom). Custom-made adapters (“lips”) used for vaping uniquely shaped device mouthpieces were fabricated in-house.

### Standard and Quality Control Material Preparation

#### Isotopically Labeled ISTD

A combined nicotine-d_4_ and menthol-d_4_ ISTD spiking solution was prepared in MeOH with concentrations of 10 mg/ml for each isotopically labeled standard. A 100-µL aliquot of this ISTD spiking solution was added to calibration standards, blanks, unknowns, and quality control (QC) samples.

#### Native Standards

A 200-mg/ml menthol stock solution in MeOH was prepared and used to make a combined nicotine and menthol stock solution with a concentration of 4 mg/ml for each analyte. We used this combined nicotine/menthol solution to prepare seven calibration standards with a concentration range of 0.010–1.000 mg/ml for both analytes and prepared with 100 µL of the ISTD spiking solution.

#### QC Materials

We prepared PG/GLY mixtures spiked with low (QCL; 2.5 mg/g) and high (QCH; 80 mg/g) nicotine and menthol concentrations to serve as matrix-based QC materials that spanned the calibration range. First, a 70/30 (v/v) PG/GLY mixture was prepared. QCL was then prepared by combining approximately 62.5 mg nicotine, 62.5 mg menthol, and 25 g of the 70/30 (v/v) PG/GLY mixture. QCH was prepared by combining approximately 2 g nicotine, 2 g menthol, and 21 g of the 70/30 (v/v) PG/GLY mixture. The QC concentrations were characterized to determine the mean concentrations and the 95th (1.96σ) and 99th (2.96σ) control limits by duplicate analysis of 19 samples of each QC level over at least 19 days. A 100-mg aliquot of each QC pool was extracted and analyzed concurrently with sample unknowns and the resulting QC data were compared to the established control limits to evaluate the validity of analyses using a set of modified Westgard rules (Westgard et al., 1981; Caudill et al., 2008).

### Aerosols/Vaping

Aerosol samples were generated according to the standard conditions described in CORESTA Recommended Method No. 81 (CORESTA, 2015) (i.e., 55 ± 0.3 ml puff volume, 3 ± 0.1 s puff duration, 30 ± 0.5 s puff interval, with a square wave puff profile) using a Cerulean CETI-8 e-cigarette vaping machine equipped with button activation switches (Cerulean, Richmond, VA, United States). We calibrated/verified the vaping machine puff volume before each use using a soap-bubble meter. Fifteen (15) puffs were taken from EVPs and the resulting aerosol was collected on individual sample CFPs; mass differences of pre- and post-vaping CFPs for a given sample [i.e., trapped total particulate matter (TPM)] were then determined gravimetrically (d = 0.00001 g). Post-vaped CFPs were removed from CFP holders and placed into 16-ml vials for extraction.

### EVP e-Liquids Sampling

For the routine analysis of e-liquids, a 100-µL sample was transferred from a given product cartridge or refill container to a 16-ml extraction vial. The masses of the liquid sample aliquots were recorded.

### Sample Preparation

Sample vials containing blanks, QCs, and post-vaped CFPs and/or liquid unknowns were spiked with 100-µL of the MeOH-based nicotine-d_4_ and menthol-d_4_ isotopically labeled ISTD spiking solution. Ten milliliters (10 ml) of MeOH were then added to each vial and all samples were placed on an orbital shaker for 10 min at 160 rpm. Aliquots of extract were transferred to GC autosampler vials for analysis.

### Instrumental Analysis

For ID-GC-MS analysis, we used an Agilent 7890A GC interfaced to an Agilent 5975C mass selective detector (MSD) MS (Agilent Technologies, Santa Clara, CA, United States) and equipped with a CTC PAL autosampler (LEAP Technologies, Carrboro, NC, United States). A 2-µL aliquot of sample extract was injected onto a 30-m Agilent J&W DB-5MS capillary column with a 0.32-mm I.D. and 1.0-µm film thickness using a 40:1 split injection. Helium was used as the carrier gas at a constant pressure of 10 psi. The injector and transfer line temperatures were set isothermally at 250 and 300°C, respectively. The initial column temperature, 150°C, was held for 1 min and then increased to 300°C at 30°C/min and held for 3 min. The MS was operated in positive electron ionization (+EI) mode and the resulting ions were analyzed using selected-ion monitoring (SIM). MS parameters were as follows: electron energy −70 eV, source temperature 230°C, quadrupole temperature 150°C, electron multiplier mode gain factor 1, mass resolution high.

We monitored one quantitation ion and two confirmation ions for each analyte and monitored an analogous isotopically labeled ISTD ion for the corresponding quantitation ion. Table 1 summarizes the SIM ions monitored, dwell times, and ion type. Data acquisition was conducted using Agilent GC/MSD ChemStation software. ChemStation data files were converted for data processing using Thermo Fisher Scientific Xcalibur™ 2.2 software.

**TABLE 1:** SIM method parameters.

Analyte	Ion (m/z)	Dwell time (msec)	Ion type
Menthol	95.1	40	Quantitation
	123.1		Confirmation 1
	138.2		Confirmation 2
	99.1		ISTD
Nicotine	162.2	40	Quantitation
	133.1		Confirmation 1
	161.1		Confirmation 2
	166.2		ISTD

### Quantitation

Calibration curves were constructed from the linear regression of the calibration standards’ analyte-to-ISTD relative response ratios versus known standard concentrations, x, with 1/x weighting. The broad calibration concentration ranges used required weighting to improve the accuracy of the lower calibrators. For aerosol analysis, results were normalized by mass of TPM, puff count, and/or total puff volume to determine analyte yields per gram of TPM (mg/g TPM), per puff (mg/puff), or per unit volume (µg/mL or mg/L), respectively. For e-liquid analysis, results were normalized by e-liquid sample mass to determine analyte levels per gram of sample (mg/g). Because the use of isotopically labeled ISTDs at the high nicotine and menthol concentrations typical of EVPs produces MS signals with increased potential for isotope contributions between native and isotopically labeled (ISTD) analogue ion channels, we implemented a correction factor (Colby and McCaman, 1979) (entered within the Xcalibur™ software quantitation method) to account for these contributions.

### Method Validation

A method validation procedure was conducted to adequately assess method performance across a broad range of EVP matrices including both hydrophilic and hydrophobic e-liquids (PG/GLY and oil-based e-liquids, respectively). The figures of merit evaluated included analytical specificity, accuracy, dynamic range, LODs, matrix effects, and precision. A description of experiments and presentation of their results are described below.

### Application: Aerosol Analysis of Products Associated With the 2019 U.S. EVALI Outbreak

The described method was used to measure nicotine and menthol in aerosol emissions from a set of EVPs associated with the 2019 U.S. EVALI outbreak. We conducted aerosol emissions testing on 141 EVP samples, including various products containing nicotine, CBD, and THC. Corresponding e-liquid analysis was not performed on this sample set. A total of 194 EVALI-related samples were received; however, 35 samples did not contain sufficient volume for analysis, and 18 samples did not produce appreciable aerosol TPM deliveries. Products that generated aerosols of less than 6.5 mg TPM per 15 puffs were considered inoperative and their data excluded. Samples were machine-vaped as described in *Aerosols/Vaping*. Due to limited sample availability, only 15 puffs per product were collected. All samples were handled following proper guidelines for the handling and analysis of potentially illicit drugs. Sample chain-of-custody was maintained and documented.

## Results and Discussion

### Figures of Merit

#### Analytical Specificity

The chromatographic specificity of the method was excellent based on baseline-resolved peaks in EVP chromatograms, the absence of interfering matrix components in representative EVP samples, and the use of isotopically labeled ISTDs. Specificity was further improved through selective detection *via* + EI and monitoring of three distinct ions (one quantitation ion and two confirmation ions) for each analyte. Retention-time monitoring and response ratios between quantitation and confirmation ions further contributed to method specificity.

#### LODs and Dynamic Range

Instrument LODs were determined based on the method described by Taylor (1987) and resulted in calculated LODs (based on final 10-ml extract concentrations) of 0.4 and 0.2 μg/ml for nicotine and menthol, respectively. For EVP liquids, these LODs correspond to 0.04 mg/ml for nicotine and 0.02 mg/ml for menthol in a 100-µL sample. For aerosols, however, TPM deliveries and nicotine/menthol concentrations vary from product-to-product, resulting in variable aerosol LODs that are based on product analyte concentrations and their respective deliveries. Despite this sample-to-sample variability, the calculated LODs are well below expected EVP nicotine and menthol concentrations and aerosol deliveries, and we thus set the limit of quantitation as the lowest calibrator and used a calibration range of 0.01–1.00 mg/ml for both analytes. Despite their addition to e-liquids at considerably high concentrations (≥0.1–5% w/w), lower analyte levels may be expected in products with low aerosol delivery (i.e., smaller sample size), as well as potential low-level products, particularly for menthol in products not obviously identified as menthol-containing.

#### Accuracy and Matrix Effects

Because the new method is intended for analyzing a variety of EVPs with e-liquids comprising PG/GLY (hydrophilic) and various hydrophobic solvents (e.g., medium chain triglycerides, vitamin E acetate, and others), we evaluated method accuracy by analyzing spiked matrix-matched samples [both hydrophilic (PG/GLY-based) and hydrophobic (oil-based products)] prepared with known concentrations of nicotine and menthol. Table 2 shows the matrix-spiked accuracy of measurements for 1) the previously described PG/GLY-based QCs, and 2) matrix-based spiked solutions with low, mid, and high analyte concentrations diluted with a pooled MeOH extract of machine-generated aerosols from commercial oil-based e-liquids. Unfortunately, no certified reference materials are available for nicotine and menthol in either hydrophobic- or hydrophilic-based products. However, our spiked matrix experiments all yielded results within 7% of their respective known concentrations across the matrix compositions and analyte concentrations tested. The accurate quantitation of these matrix-based samples using a solvent-based calibration curve demonstrated the absence of matrix effects in both hydrophilic and hydrophobic matrices (Table 2). These results confirm the applicability and use of the described method for accurate measurements of nicotine and menthol in EVP liquids and/or aerosol samples of varying matrix composition.

**TABLE 2:** Method accuracy (% error) in hydrophilic (PG/GLY) and hydrophobic (oil-based) matrix-based spiked solutions.

Analyte	Hydrophilic (PG/GLY; *n* = 5)		Hydrophobic (oil-based; *n* = 6)		
	2.5 mg/g	80 mg/g	0.025 mg/ml	0.125 mg/ml	0.750 mg/ml
Menthol	−3.0%	−1.9%	−2.5%	0.0%	−1.6%
Nicotine	4.8%	7.0%	3.6%	5.4%	5.6%

#### Precision

Method precision was assessed as repeatability and intermediate precision in terms of percent relative standard deviations (%RSDs). Precision was assessed from the duplicate analysis of 19 sample sets of the QC materials (2.5 and 80 mg/g) over 19 different days. Repeatability was calculated as within-run variation of duplicates, while intermediate precision was calculated as the between-run, or total, variation. We observed excellent method repeatability (<0.5%) and intermediate precision (<3%) at both low and high concentrations for both analytes.

### Application: Aerosol Analysis of Products Associated With the 2019 U.S. EVALI Outbreak

To demonstrate “fit for purpose,” we applied the described method to analyze aerosol samples from the 2019 U.S. EVALI outbreak (Blount et al., 2020). The method performed well for both hydrophilic (PG/GLY-based) and hydrophobic (e.g., oil-based medium chain triglycerides, vitamin E acetate, and others) e-liquids, demonstrating its versatility. Nicotine and menthol were detected at quantifiable concentrations in 28% (39 samples) and 11% (16 samples) of the 141 samples analyzed, respectively. Nicotine was only detected in PG/GLY-based products at concentrations ranging between 2.19 and 59.5 mg/g of aerosol TPM (0.2–6.0% nicotine) and was not detected in any of the THC or oil-based products. Of the 39 nicotine product samples, 15 also contained menthol with concentrations that ranged between 1.09 and 10.69 mg/g of aerosol TPM (0.1–1.1% menthol). Menthol was detected in only one THC-containing oil-based product at a concentration of 2.95 mg/g of aerosol (0.3% menthol). No nicotine was detected above LOD in any samples containing vitamin E acetate (the likely causal toxicant); thus, the EVALI outbreak is more closely associated with THC products than nicotine products (Blount et al., 2020).

## Conclusion

The described ID-GC-MS method provides accurate and precise quantitation of nicotine and menthol concentrations in hydrophilic (PG/GLY-based) and hydrophobic (oil-based) e-liquids and machine-generated aerosols of EVPs. Application of the method for EVPs associated with the 2019 EVALI outbreak and the detection of nicotine and menthol in a portion of these products demonstrate that the method is fit-for-purpose.

## Data Availability

The raw data supporting the conclusions of this article will be made available by the authors, without undue reservation.
